# New approach to modulate retinal cellular toxic effects of high glucose using marine epa and dha

**DOI:** 10.1186/1743-7075-8-39

**Published:** 2011-06-16

**Authors:** Mélody Dutot, Violaine de la Tourrette, Roxane Fagon, Patrice Rat

**Affiliations:** 1 Yslab, Quimper, France; 2 Laboratoire de Chimie et Toxicologie Analytique et Cellulaire, Faculté de Pharmacie, Université Paris Descartes, Paris, France

**Keywords:** Glucose, Inflammation, Omega-3, Oxidative stress, Caveolin-1

## Abstract

**Background:**

Protective effects of omega-3 fatty acids against cellular damages of high glucose were studied on retinal pigmented epithelial (RPE) cells.

**Methods:**

Retinal epithelial cells were incubated with omega-3 marine oils rich in EPA and DHA and then with high glucose (25 mM) for 48 hours. Cellular responses were compared to normal glucose (5 mM): intracellular redox status, reactive oxygen species (ROS), mitochondrial succinate deshydrogenase activity, inflammatory cytokines release and caveolin-1 expression were evaluated using microplate cytometry, ELISA and flow cytometry techniques. Fatty acids incorporation in retinal cell membranes was analysed using chromatography.

**Results:**

Preincubation of the cells with fish oil decreased ROS overproduction, mitochondrial alterations and TNFα release. These protective effects could be attributed to an increase in caveolin-1 expression induced by marine oil.

**Conclusion:**

Marine formulations rich in omega-3 fatty acids represent a promising therapeutic approach for diabetic retinopathy.

## Background

Diabetic retinopathy is the most common diabetic eye disease and a leading cause of blindness in adults. It is estimated that in 2002, diabetic retinopathy accounted for about 5% of world blindness, representing almost 5 million blind people [1].

Sustained hyperglycemia appears to be the major contributor to the development of this multifactorial disease [2]. The hallmarks of diabetic retinopathy include blood-retinal barrier breakdown [3]. The retinal pigmented epithelial (RPE) cells constitute the outer blood-retinal barrier. Oxidative stress plays a central role in the pathogenesis of diabetic retinopathy [4-6]. In addition to oxidative stress, inflammation is implicated in diabetic retinopathy. Retinal leukostasis increases within days of developing diabetes and correlates with the increased expression of retinal intercellular adhesion molecule-1 and CD18 [7-9]. Therefore, antioxidants have been found to inhibit the development of inflammatory changes in retinas of diabetic animals [5]. Omega-3 fatty acids (mainly EPA and DHA) possess antioxidant and anti-inflammatory activities. They incorporate into retinal cell membranes [10,11] and could be proposed as potential preventive therapies to diabetic patients.

The aim of this study was to modulate the side effects of high glucose using marine omega-3-rich oil on RPE cells.

## Methods

### Reagents

Omega-3-rich oil (fish YS-2636) was provided from Yslab (Quimper, France). Composition of oils is summarized in table 1. Chemicals, cell culture reagents and fluorescent dyes were purchased from Sigma Aldrich, Eurobio (Les Ulis, France) and Invitrogen (Cergy Pontoise, France), respectively.

**Table 1:** EPA and DHA (%) and tocopherol (mg/g) composition of tested oil

	Fish YS-2636
C20:5 w3 EPA	36

C22:6 w6 DHA	26

Mixed Tocopherol	3.6

EPA: eicosapentenoic acid

DHA: docosahexaenoic acid

### Experimental procedures

#### Cell Culture

A human retinal pigmented epithelial cell line (ARPE-19, ATCC CRL2302) was cultured under standard conditions in DMEM containing 1 g/L of glucose supplemented with 10% foetal calf serum, 2 mM L-glutamine, 50 IU/ml penicillin and 50 IU/ml streptomycin. The medium was changed every 2 days. Confluent cultures were removed by trypsin incubation, and then the cells were counted. They were seeded into 96-well culture microplates at a density of 80,000 cells/well and kept at 37°C for 24 hours.

#### Incubation protocols

Whenever cells reached 80% confluency, the culture medium was removed and the cells were exposed to neat fish oil for 15 minutes. The cells were rinsed with phosphate buffer and incubated in culture medium for 24 hours [12-14]. The cells were then incubated with high glucose (4.5 g/L) for 48 hours.

#### Metabolic activity

Alamar Blue assay uses resazurine, a visible blue fluorogene probe, which is reduced to a red fluorescent compound (resorufin) by cellular redox enzymes. Cells were incubated with resazurine solution (0.1 mg/ml) containing culture medium supplemented with 2.5% foetal calf serum. After a 6-h incubation time at 37°C in moist atmosphere with 5% CO_2_, resorufin fluorometric signal (λexc = 535 nm, λem = 600 nm) was measured using a microplate fluorometer (Safire, Tecan, France).

#### Reactive oxygen species (ROS) production evaluation

ROS were detected with the 2',7'-dichlorofluorescein diacetate probe. Once inside the cell, this probe is cleaved by endogenous esterases and can no longer pass out of the cell. The de-esterified product becomes the fluorescent compound 2',7'-dichlorofluorescein after oxidation by reactive oxygen species. Cells were incubated for 20 minutes with a 20 μM DCFH-DA solution, fluorescence detection (λexc = 485 nm, λem = 535 nm) was undertaken with a microplate fluorometer (Safire, Tecan, France).

#### Mitochondrial dehydrogenase activity

The activity of mitochondrial deshydrogenase was measured using the MTT probe. The assay is based on the reduction of yellow tetrazolium salt MTT onto water-insoluble purple formazan salt by viable cells. Cells were incubated with a MTT solution at 0.5 mg/mL for 3 hours and rinsed with phosphate buffer. Formazan salt was solubilized in DMSO and absorbance detection (λabs = 540 nm) was undertaken with a microplate colorimeter (Safire, Tecan, France).

#### Cytokines release

The release of TNFα in cell supernatants was determined by ELISA. After glucose incubation, cell supernatants were harvested and stored at -20°C until use for cytokines measurements. The quantity of released cytokines was measured according to the manufacturer's instructions (R&D Systems, Lille, France).

#### Fatty acid composition of RPE cells

The cells were centrifuged for 5 minutes at 1000 g. The cell pellets were suspended in 1 mL of 0.1 M sucrose-10 mM Tris buffer (pH 7.4) at 4°C. They were then lysed after five freeze-thaw cycles and a cold sonication for 30 to 60 seconds. Cell membranes were isolated by several ultracentrifugations. Total lipids were extracted, and membrane fatty acids were analyzed and quantified by high-pressure liquid chromatography, as previously described [15].

#### Immunofluorescence for caveolin-1 expression

The culture cells were harvested with trypsin-EDTA, pelleted, washed twice in PBS, and fixed in paraformaldehyde. After permeabilization, the cells were incubated with anti-caveolin-1 antibody for 45 minutes. After wash, the cells were incubated with fluorescein isothiocyanate (FITC)-conjugated secondary antibody for 45 minutes. Flow cytometric quantitation of fluorescence was measured using C6-Flow^® ^cytometer (Accuri, France).

### Data Analyses

Data in the text and graphs are shown as fluorescence percentage of the control (data are normalized to cell number), and statistical analyses was performed using a one-way ANOVA followed by a Dunnet's test (α risk = 0.05). Each test was performed in triplicate.

## Results

### Cell viability: metabolic activity

**Figure 1:**
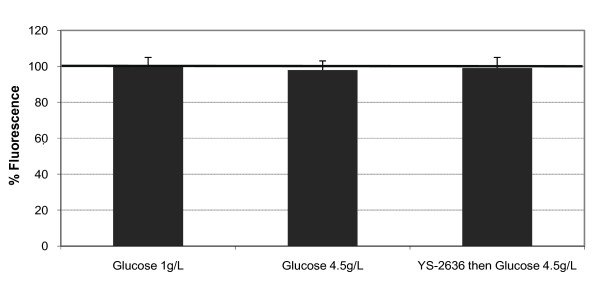
**Metabolic activity of RPE and Müller cells**. The cells were incubated with YS-2636 fish oil for 15 minutes. After the cells were rinsed with phosphate buffer, the cells were incubated with high glucose (4.5 g/L) for 48 hours. Normal glucose (1 g/L) was used as a cell control.

As shown in Figure 1, high glucose didn't alter the cell viability of RPE cells. Tested marine oil was safe for RPE cells.

### Production of ROS

**Figure 2:**
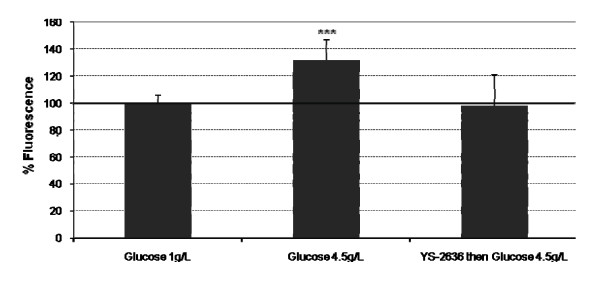
**Production of reactive oxygen species by RPE cells and Müller cells**. The cells were incubated with YS-2636 fish oil for 15 minutes. After the cells were rinsed with phosphate buffer, the cells were incubated with high glucose (4.5 g/L) for 48 hours. Normal glucose (1 g/L) was used as a cell control. ***p < 0.001.

As shown in Figure 2, high glucose induced a significant overproduction of ROS in RPE cells (+32%) compared to normal glucose. Preincubation with fish YS-2636 oil totally inhibited ROS high glucose-induced production in RPE cells, as ROS production was not significantly different from normal glucose (98% compared to normal glucose).

### Mitochondrial dehydrogenase activity

**Figure 3:**
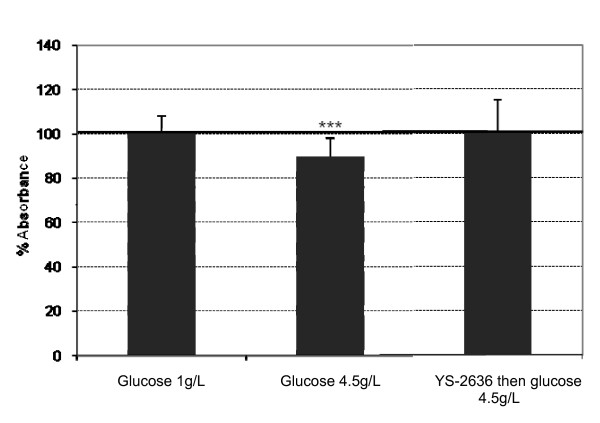
**Mitochondrial dehydrogenase activity**. The cells were incubated with YS-2636 fish oil for 15 minutes. After the cells were rinsed with phosphate buffer, the cells were incubated with high glucose (4.5 g/L) for 48 hours. Normal glucose (1 g/L) was used as a cell control. ***p < 0.001.

As shown in Figure 3, high glucose induced a significant decrease in mitochondrial dehydrogenase activity in RPE cells (-10%) compared to normal glucose. Fish YS-2636 oil protected cells against high glucose-induced decrease in mitochondrial dehydrogenase activity on RPE cells.

### Cytokines release

**Figure 4:**
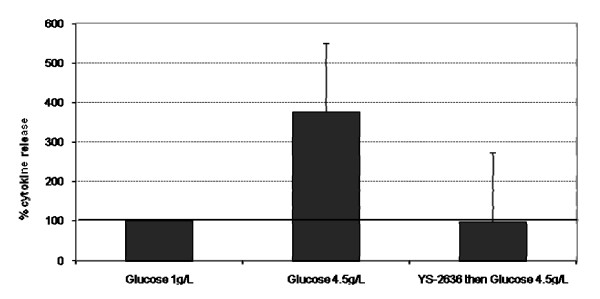
**Release of TNFα proinflammatory cytokines**. The cells were incubated YS-2636 fish oil for 15 minutes. After the cells were rinsed with phosphate buffer, the cells were incubated with high glucose (4.5 g/L) for 48 hours. Normal glucose (1 g/L) was used as a cell control.

As shown in Figure 4, high glucose induced TNFα release in RPE cells. YS-2636 inhibited TNFα release induced by high glucose.

### Fatty acid composition of RPE cells

As shown in table 2 fish YS-2636 oil increased EPA (from 1.58% in control cells to 4.10% in cells incubated with YS-2636) and decreased DHA (from 11.60% in control cells to 6.65% in cells incubated with YS-2636).

**Table 2:** Effect of fish oil incubation on the fatty acid composition (%) of RPE cells

	Control cells	Cells incubated with YS-2636 oil
**20:4w6 AA**	11.60	6.65 *(p < 0.001)*
**20:5w3 EPA**	1.58	4.10 *(p < 0.001)*
**22:6w3 DHA**	5.32	5.40

**PUFA** **PUFA w6**	29.77 20.24	39.41 *(p < 0.001)* 26.27 *(p < 0.005)*
**PUFA w3**	9.54	13.14 *(p < 0.005)*

AA: arachidonic acid

EPA: eicosapentenoic acid

DHA: docosahexaenoic acid

PUFA: polyunsaturated fatty acid

### Caveolin-1 expression

**Figure 5:**
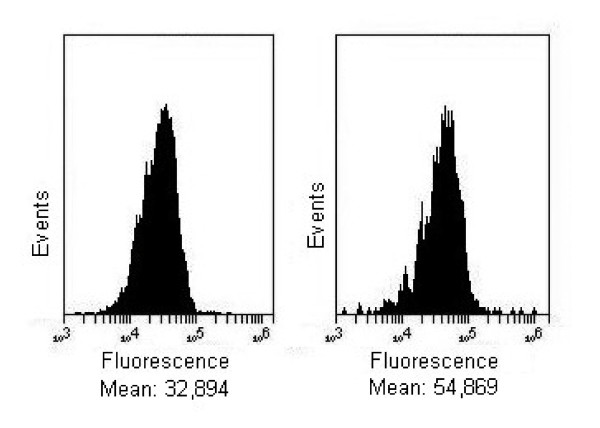
**Evaluation of caveolin-1 expression**. The RPE cells were incubated with culture medium (left chart) or YS-2636 oil (right chart) for 15 minutes. After the cells were rinsed with phosphate buffer, the cells were incubated with culture medium for 24 hours and fixed in PFA. The expression of caveolin-1 was quantified using flow cytometry. Figures are representative of three independent experiments.

As shown in Figure 5, fish YS-2636 oil increased the expression of caveolin-1 in RPE cells (+60% compared to control).

## Discussion

Prolonged exposure of the cells to high glucose is shown to cause both acute and reversible changes in cellular metabolism [4]. We confirmed that high glucose induced ROS overproduction on RPE cells, in accordance with a previous study [16]. Du et al. observed that high glucose-induced oxidative stress came predominantly from mitochondria [17]. Mitochondria is the organelle where most of radicals are produced. Electron leak to oxygen through respiratory chain complexes can generate superoxide anion [18]. At the same time, the mitochondrial respiratory chain is an important target for the damaging effects of ROS [19]. In RPE cells, we observed ROS overproduction and a slight decrease in mitochondrial dehydrogenase activity. In our model (48 hours in high glucose), ROS overproduction may not be generated only by the mitochondrial respiratory chain but also through NADPH oxidase for example [20]. There is evidence that the release of TNFα induced by high glucose in vitro may be mediated by ROS [6]. TNFα, overexpressed in tissues of diabetic patients [21,22], has been implicated in insulin resistance. Interestingly, in our model, TNFα was released by RPE cells, associated with ROS overproduction.

An important role for DHA within the retina is suggested by its high levels (8-20% of total retinal fatty acids in humans) in this tissue. Therefore, we tested the protective effects of fish oil rich in DHA (26% of total fatty acids). This marine oil was able to prevent high glucose from being toxic to retinal cells. It also contains EPA (36% of total fatty acids); EPA is the omega-3 homologue of arachidonic acid and competes with arachidonic acid for metabolism by COX enzymes. Arachidonic acid is at the origin of inflammatory mediators whereas EPA is at the origin of anti-inflammatory mediators. An increase in EPA can lead to a decrease in arachidonic acid [12] and then to a decrease in the inflammatory response. We observed that incubation of RPE cells with fish oil increased EPA and decreased arachidonic acid in RPE cell membranes, total omega-3 polyunsaturated fatty acids being increased from 9.54% to 13.14%. When the RPE is under a state of oxidative stress, a molecule called Neuroprotectin D-1 is synthesized by the RPE. Neuroprotectin D-1 is derived from DHA, and provides anti-inflammatory and cellular protection to the retina. This DHA derived molecule protects the retinal cells from damage and consequently cellular death [23,24]. Resolvin E1 is derived from EPA and is another omega-3-derived counterregulatory anti-inflammatory lipid mediator [25]. EPA and DHA, both precursors of anti-inflammatory lipid mediators, are potent anti-inflammatory agents.

A recent study concluded that fish oil, containing EPA and DHA, improved glucose transporter expressions decreased by insulin [26]. Homeostasis of glucose by insulin involves stimulation of glucose uptake by translocation of glucose transporter from intracellular pool to the caveolar membrane system [27]. In view of the fact that omega-3-rich oils are able to modulate glucose transporter expression and that glucose transporter translocation is linked to caveolar membrane system, we studied the expression of caveolin-1, an indispensable protein for both the structure and function of caveolae, after incubation of retinal cells with omega-3-rich oil. We observed that the marine omega-3-rich oil we tested increased caveolin-1 expression in RPE cells. Caveolins can promote signaling *via *enhanced receptor-effector coupling or enhanced receptor affinity when caveolins are up-regulated or overexpressed [28]. Marine omega-3-rich oils can up-regulate caveolin-1 expression leading to glucose transporters overexpression in caveolae. Further studies are needed to better understand the underlying mechanisms.

In conclusion, marine EPA and DHA seem beneficial and essential in the modulation of high glucose toxicity for RPE cells. In vivo studies are needed to confirm the therapeutic effects of this oil.

## Competing interests

The authors declare that they have no competing interests.

## Authors' contributions

VT carried out the ELISA studies and participated in the design of the study. PR and RF participated in the design of the study and participated in its coordination. All authors read and approved the final manuscript.
